# Prognostic factors of surgical management for heart failure patients with reduced left ventricular ejection fraction

**DOI:** 10.1016/j.heliyon.2024.e26552

**Published:** 2024-02-20

**Authors:** Mahmoud Yousef Ibrahim Abuharb, Liu Kaiwen, Huang Zhuhui, Zhang Kui, Zheng Jubing, Song Yue, Li Yang, Liu Taoshuai, Dong Ran

**Affiliations:** Capital Medical University Affiliated Anzhen Hospital, Cardiovascular Surgery Department, 100029, China, Beijing

**Keywords:** Heart failure, LVEF, Retrospective, OPCAB, ONCAB, Heart function

## Abstract

**Objectives:**

There are many available pharmaceutical and surgical management for Coronary artery disease (CAD) patients. However, coronary artery bypass grafting (CABG) is the preferred treatment modality for CAD patients with low ejection fraction (EF) in view of the more favorable outcomes. This study aimed to determine the associated factors of poor outcomes post-CABG for heart failure patients with reduced left ventricular ejection fraction who underwent on-pump and off-pump CABG.

**Methods:**

A retrospective review of CAD patients who underwent isolated on-pump CABG (ONCAB) or off-pump CABG (OPCAB) in Beijing Anzhen Hospital Affiliated with Capital Medical University from January 2013 to March 2021. Only those with confirmed reduced left ventricular ejection fraction (LVEF) ≤40% on preoperative echocardiography were included. By analyzing the clinical and surgical data, postoperative mortality and morbidity, as well as major cardiovascular and cerebrovascular adverse events (MACCE) as endpoints, certain risk factors of the postoperative complications were identified.

**Results:**

Out of the 500 patients, 64 developed MACCE, of which 14 (13.6%) occurred in the ONCAB group and 50 (14.0%) in the OPCAB group. Univariate COX regression analysis showed that age ≥65 years, history of diabetes, and preoperative renal insufficiency were independent risk factors for postoperative primary endpoint events in CAD patients with heart failure with reduced ejection fraction (HFrEF). Following the multivariate COX regression analysis, in addition to the above three risk factors, a history of previous percutaneous coronary angiography (PCI) intervention was also a risk factor for the occurrence of the primary endpoints post-CABG.

**Conclusion:**

Based on the analysis, significant predictors of post-CABG MACCE in patients with HFrEF included being older than 65 years old, having diabetes, preoperative renal insufficiency, and having previous PCI.

## Abbreviations

**CABG**: Coronary artery bypass graft
**CPB**: Cardiopulmonary bypass
**LVEF**: Left ventricular ejection fraction
**LVEDD**: Left ventricular end-diastolic diameter
**EF**: Ejection fraction
**ECHO**: Echocardiography
**ONCAB**: On-pump coronary artery bypass
**OPCAB**: Off-pump coronary artery bypass
**GFR**: Glomerular filtration rate
**CK-MB**: Creatine kinase-myoglobin binding
**Cr**: Serum creatinine
**TTE**: Transthoracic echocardiography
**LV**: Left ventricle
**RV**: Right ventricle
**LA**: Left atrium
**RA**: Right atrium
**RVSF**: Right ventricular systolic function
**SD**: Standard deviation
**RCA**: Right coronary artery
**LAD**: Left anterior descending artery
**LCX**: Left circumflex artery
**LIMA**: Left internal mammary artery
**MACCE**: Major adverse cardiovascular and cerebrovascular events

## 1 Introduction

Coronary artery disease (CAD) is a disease that can cause severe effects on the health and well-being of patients. Among the Chinese population, the morbidity and mortality of CAD are increasing in trend [1]. Coronary atherosclerotic heart disease, or CAD is the commonest cause of heart failure (HF) [2]. The mortality rate of CAD is higher when HF is present [3]. According to the China Cardiovascular Disease Report 2018, there are as high as 11 million patients with ischemic cardiomyopathy and 4.5 million patients with HF in the country, with a continuous rise in prevalence and mortality. Thus, timely prevention and appropriate treatment are urgently needed [4].

In 2021, the European Society of Cardiology (ESC) established a new classification according to the value of left ventricular ejection fraction (LVEF). The Heart Failure with Reduced Ejection Fraction (HFrEF) classification included Heart Failure with Mildly Reduced Ejection Fraction (HFmrEF) and Heart Failure with Preserved Ejection Fraction (HFpEF). HFrEF is defined as LVEF ≤40% with symptoms and/or signs of HF [5]. In the guidelines for myocardial revascularization issued by the ESC in 2018, the European Association of Cardiothoracic Surgery (EACTS) proposed that among CAD patients with HF and severe left ventricular systolic dysfunction who are suitable for myocardial revascularization, coronary artery bypass grafting surgery (CABG) should be offered as the preferred revascularization strategy [6].

For CABG, there are two basic methods, i.e. isolated on-pump CABG (ONCAB) or off-pump CABG (OPCAB), each with its advantages and disadvantages. In the literature, even though ONCAB is associated with better revascularization outcomes, OPCAB is associated with a lower rate of postoperative morbidity and mortality, especially among high-risk patients [7]. In clinical practice, the left ventricular function is instrumental in the outcome of CABG surgery. Therefore, prudent choice and planning of surgical methods for CAD patients with LVEF less than 40% is important.

## 2 Methods and patients

A total of 500 patients with HFrEF were enrolled continuously in this study, with a median follow-up time of 924 days. The 459 patients were categorized as the MACCE event group and no MACCE event group.

### 2.1 Operative techniques

For the CABG operation, the following steps were performed. After placing the patient placed in the supine position, general anesthesia with endotracheal intubation was conducted. After the median sternotomy was done, the decision to perform OPCAB or ONCAB was made based on the patient's condition and the surgeon's experience, since the fact that till the moment there are no guidelines regarding patient selection, so the surgeons made their choice for surgical procedure totally based on the patients' preoperative clinical status where patients with more comorbidities and higher risk factors like lung failure, kidney failure, stroke, advanced age and those whom were clinically unstable in general were referred to ONCAB. And it worth to mention that among the patients who were initially randomized to the OPCAB group, 20 were converted into CPB during CABG. Firstly, the internal thoracic artery was harvested by the skeletonized or pedicled technique [8], and the left internal thoracic artery was grafted to the left anterior descending artery. After that, the great saphenous vein and radial artery were obtained by the open technique. Following that, the circumflex branch and the right coronary artery were sequentially anastomosed.

In this study, all patients received anatomically complete revascularization, i.e. coronary angiography showed at least one lesion and the revascularization was performed for vessels with a diameter of more than 1.5 mm and a stenosis of ≥50%. The transient blood flow measurement technique was performed to assess the quality of graft anastomosis.

### 2.2 Echocardiography

In clinical practice, LVEF is commonly assessed by ECHO. Pre- and postoperative transthoracic ECHO (TTE) was conducted on all patients in this study. In addition, they also received regular monitoring of their LV functions throughout the follow-up period.

Severe LV systolic dysfunction is defined as EF ≤ 40%. An absolute increase of ≥5% in the baseline preoperative LVEF represented an ECHO Improvement in LVEF. In contrast, a decrease of >5% indicated worsened LVEF. Lastly, any postoperative EF that was within ±5% of the baseline preoperative value would be categorized as unchanged LVEF [9].

### 2.3 Follow-up

Patients were followed up at 3 and 6 months post CABG, and subsequently every 6 months thereafter. The median follow-up time among the patients in this study was 924 days. During the follow-up, the general condition of the patients was assessed. The presence of MACCE, i.e. all-cause death, myocardial infarction, HF, cerebral infarction, revascularization, and cardiac-related rehospitalization was also evaluated. In addition to approaching the patients at the outpatient clinics, telephone calls and local communication applications were also used to follow up with the patients. Thus, only a small number of patients (n = 41, 8.2%) were lost to follow-up.

### 2.4 Statistical analysis

A comparison of continuous variables was performed using two independent samples T-test or the Mann-Whitney *U* test. Any variables with p-value <0.20 in the univariate COX regression analysis were included in the multivariate COX regression analysis. In addition, the Kaplan-Meier (K-M) method was used to determine the cumulative survival rate and the factors affecting the outcome were screened by the Lasso method [10]. Statistical analysis was performed using the R language (Version 4.0.2, http://www.Rproject.org). All tests were two-sided and p-value <0.05 was considered statistically significant.

## 3 Results

A total of 41 patients (8.2%) were lost to follow-up. Among the 459 patients who completed the follow-up, 64 patients (13.9%) developed MACCE. No significant differences were detected between the two groups at baseline and endpoint (Table 1).

1. Univariate COX regression

**Table 1:** Baseline characteristics and primary endpoints of patients.

	Overall	No MACCE	MACCE	p-value
	N = 459	N = 395	N = 64	
Female sex	78 (17.0)	60 (15.2)	18 (28.1)	0.017
Age（years）	61.45 (9.57)	61.08 (9.56)	63.73 (9.42)	0.039
Age≥65 years	197 (42.9)	159 (40.3)	38 (59.4)	0.006
Inpatient time	18.25 (8.70)	17.97 (8.65)	19.98 (8.87)	0.086
BMI	25.62 (3.16)	25.69 (3.21)	25.22 (2.83)	0.269
Hypertension	255 (55.6)	220 (55.7)	35 (54.7)	0.988
Diabetes	219 (47.7)	177 (44.8)	42 (65.6)	0.003
Smoking	243 (52.9)	213 (53.9)	30 (46.9)	0.361
Alcohol	113 (24.6)	102 (25.8)	11 (17.2)	0.183
Hyperlipidemia	136 (29.6)	116 (29.4)	20 (31.2)	0.874
Renal insufficiency	61 (13.3)	45 (11.4)	16 (25.0)	0.006
Lung disease	18 (3.9)	16 (4.1)	2 (3.1)	0.995
Cerebrovascular disease	73 (15.9)	59 (14.9)	14 (21.9)	0.221
Previous PCI	55 (12.0)	43 (10.9)	12 (18.8)	0.112
MI	192 (41.8)	165 (41.8)	27 (42.2)	1.000
NYHA classes				0.701
I	6 (1.3)	6 (1.5)	0 (0.0)	
II	148 (32.2)	125 (31.6)	23 (35.9)	
III	221 (48.1)	192 (48.6)	29 (45.3)	
IV	84 (18.3)	72 (18.2)	12 (18.8)	
LVEF（%）	35.75 (3.28)	35.84 (3.20)	35.20 (3.70)	0.153
LVEDD（mm）	57.06 (6.54)	57.18 (6.52)	56.31 (6.71)	0.323
LVEF≤30%	40 (8.7)	31 (7.8)	9 (14.1)	0.163
LVEDD measures				0.494
<56 mm	171 (37.3)	143 (36.2)	28 (43.8)	
56∼60 mm	166 (36.2)	146 (37.0)	20 (31.2)	
>60 mm	122 (26.6)	106 (26.8)	16 (25.0)	
OPCAB	356 (77.6)	306 (77.5)	50 (78.1)	1.000

BMI; Body mass index, PCI; Percutaneous coronary intervention, MI; myocardial infarction, NYHA; New York heart association, LVEF; Left ventricular ejection fraction, LVEDD; Left ventricular end-diastolic diameter, OPCAB; off-pump coronary artery bypass grafting.

Using univariate COX regression analysis in all patients, it was found that the primary endpoint was significantly correlated with multiple predictors and these are: gender, age, history of diabetes, history of alcohol consumption, preoperative renal insufficiency, history of cerebrovascular disease, previous PCI operations, preoperative LVEF, and preoperative LVEDD as shown in Table 2.

**Table 2:** Univariate COX regression analysis of patients.

	Overall （N = 459）	No MACCE （N = 395）	MACCE （N = 64）	HR [95%CI]	P value
Female sex	78 ± 17.0	60 ± 15.2	18 ± 28.1	2.066 [1.200; 3.558]	0.009
Age（years）	61.45 ± 9.57	61.08 ± 9.56	63.73 ± 9.42	1.031 [1.001; 1.061]	0.043
Age ≥65 years	197 (42.9)	159 (40.3)	38 (59.4)	2.110 [1.282; 3.473]	0.003
BMI	25.62 ± 3.16	25.69 ± 3.21	25.22 ± 2.83	0.956 [0.888; 1.029]	0.229
Hypertension	255 (55.6)	220 (55.7)	35 (54.7)	0.980 [0.599; 1.605]	0.937
Diabetes	219 (47.7)	177 (44.8)	42 (65.6)	2.280 [1.365; 3.809]	0.002
Smoking	243 (52.9)	213 (53.9)	30 (46.9)	0.750 [0.459; 1.227]	0.252
Alcohol	113 (24.6)	102 (25.8)	11 (17.2)	0.622 [0.324; 1.194]	0.154
Hyperlipidemia	136 (29.6)	116 (29.4)	20 (31.2)	1.126 [0.665; 1.909]	0.658
Renal insufficiency	61 (13.3)	45 (11.4)	16 (25.0)	2.514 [1.412; 4.478]	0.002
Lung disease	18 (3.9)	16 (4.1)	2 (3.1)	0.764 [0.187; 3.124]	0.708
Cerebrovascular disease	73 (15.9)	59 (14.9)	14 (21.9)	1.609 [0.886; 2.920]	0.118
Previous PCI	55 (12.0)	43 (10.9)	12 (18.8)	1.685 [0.898; 3.161]	0.104
Myocardial infarction	192 (41.8)	165 (41.8)	27 (42.2)	0.977 [0.597; 1.599]	0.927
Heart function NYHA class				0.982 [0.706; 1.366]	0.915
I	6 (1.3)	6 (1.5)	0 (0.0)		
II	148 (32.2)	125 (31.6)	23 (35.9)		
III	221 (48.1)	192 (48.6)	29 (45.3)		
IV	84 (18.3)	72 (18.2)	12 (18.8)		
Number of grafts	3.37 ± 0.93	3.38 ± 0.87	3.27 ± 1.25	0.883 [0.631; 1.235]	0.468
LVEF（%）	35.75 ± 3.28	35.84 ± 3.20	35.20 ± 3.70	0.954 [0.889; 1.023]	0.183
LVEDD（mm）	57.06 (6.54)	57.18 (6.52)	56.31 (6.71)	0.982 [0.946; 1.019]	0.335
OPCABG	356 (77.6)	306 (77.5)	50 (78.1)	1.071 [0.586; 1.956]	0.823

HR: Hazard ratio, CI: Confidence interval, BMI: Body mass index, PCI: Percutaneous coronary intervention, NYHA: New York Heart Association, LVEF: Left ventricular ejection fraction, LVE DD: Left ventricular end-diastolic diameter, LVESD: Left ventricular end-systolic diameter, OPCABG: Off-pump coronary artery bypass grafting.

All variables from univariate Cox Regression analysis (gender, age, history of diabetes, history of alcohol consumption, preoperative renal insufficiency, history of cerebrovascular disease, previous PCI operations, preoperative LVEF, and preoperative LVEDD) were included in the multivariate COX regression analysis.

2. Multivariate COX regression

Multivariate COX regression analysis showed that age ≥65 years, history of diabetes, preoperative renal insufficiency, and previous PCI were independent predictors of primary endpoints post CABG among CAD patients complicated with HFrEF. The hazard ratio (HR), 95% confidence interval (CI), and p-value of each variable in the multivariate COX analysis results are shown in Table 3.

**Table 3:** The results of multivariate COX regression analysis of patients.

	Overall （N = 459）	No MACCE （N = 395）	MACCE （N = 64）	HR[95%CI]	P value
Age ≥65 years rowhead	197 (42.9)	159 (40.3)	38 (59.4)	1.981 [1.172; 3.348]	0.011
Diabetes rowhead	219 (47.7)	177 (44.8)	42 (65.6)	2.206 [1.307; 3.723]	0.003
Renal insufficiency rowhead	61 (13.3)	45 (11.4)	16 (25.0)	2.134 [1.198; 3.802]	0.010
Previous PCI rowhead	55 (12.0)	43 (10.9)	12 (18.8)	1.893 [1.046; 3.424]	0.035

## 4 Discussion

Based on the multivariate regression analysis, four predictors were significantly associated with the primary endpoints, namely age ≥65 years, diabetes, renal insufficiency, and previous PCI.

In the literature, the Surgical Treatment of Ischemic Heart Failure (STICH) study reported the outcomes of CABG in patients with LVEF <35%. The 10-year follow-up (STITCHES) results showed that CABG was associated with better outcomes compared to guideline-directed medical therapy (GDMT) [11]. And the 30-day mortality rate among the trial patients was 5.1%. Similarly, in a study by Topkara et al. using the patient database in New York State, patients with LVEF <20% reported five times higher in-hospital mortality rate and other complications compared to patients with LVEF >40% [12].

In the study, Omer S et al. reported that a decrease in LVEF was associated with a higher occurrence of postoperative complications. Furthermore, it was associated with a lower 10-year survival after CABG. Overall, it was found that patients who suffered from post-CABG complications had a decreased 10-year survival regardless of their LVEF [13].

There are several limitations to this study. Firstly, as a retrospective study, selection bias was unavoidable. Thus, a prospective cohort study is warranted to establish the research findings. Next, the study sample size was rather small, and future studies should include more HFrEF patients undergoing CABG to further validate the predictive performance of post-CABG mortality and morbidity indicators. In addition, LV volumes that can reflect LV enlargement better than LV inner diameters were not included in this study due to the lack of data. Lastly, literature findings have shown that chamber quantification parameters on cardiac magnetic resonance imaging can be more accurate and convey a higher predictive power for the outcome. Nevertheless, we did not manage to obtain sufficient data for analysis.

## 5 Conclusion

Four predictors were significantly associated with the primary endpoints, namely age ≥65 years, diabetes, renal insufficiency, and previous PCI. While the surgical treatment wasn't a risk factor for adverse outcomes.

## Ethics statement

Ethical approval was obtained from the Institutional Ethics Committee of Beijing Anzhen Hospital. The patients provided their written informed consent to participate in this study, ethics approval number (A20239871).

## Consent for publication

Written informed consent was obtained from all the patients. A copy of the written consent is available for review.

## Availability of data and material

The raw data supporting the conclusion of this article will be made available by the authors, without undue reservation.

## Funding

This study was supported by a grant from the 10.13039/501100001809National Natural Science Foundation of China (81770412), 10.13039/501100009592Beijing Municipal Science and Technology Commission (Z151100003915084), as well as the Beijing Municipal Health Commission Capital Health Development Scientific Research Project (Shoufa 2020-2Z-2067).

## CRediT authorship contribution statement

**Mahmoud Yousef Ibrahim Abuharb:** Writing – review & editing. **Liu Kaiwen:** Formal analysis. **Huang Zhuhui:** Data curation. **Zhang Kui:** Data curation. **Zheng Jubing:** Supervision. **Song Yue:** Resources. **Li Yang:** Visualization. **Liu Taoshuai:** Resources. **Dong Ran:** Supervision, Project administration.

## Declaration of competing interest

The authors declare that they have no known competing financial interests or personal relationships that could have appeared to influence the work reported in this paper.
